# Made to feel different: Families perspectives on external responses to autism and the impacts on family well-being and relationships

**DOI:** 10.1177/13623613231221684

**Published:** 2024-01-19

**Authors:** Sebastian Trew

**Affiliations:** Australian Catholic University, Australia

**Keywords:** autism spectrum disorders, environmental factors, family functioning and support, health services, school-age children, social services

## Abstract

This study aimed to investigate the impact of external factors on the experiences and family relationships of autistic adolescents and their families. A qualitative approach was used, with in-depth interviews conducted with 30 participants, including autistic adolescents, siblings and parents from within families with an autistic adolescent family member. The findings suggest that external factors significantly influence the well-being of autistic adolescents and their families. The school environment was identified as a significant factor, with sensory overload, bullying and adverse interactions with teachers having a negative impact on the mental health and quality of family relationships for participants. In addition, the study highlighted that isolation and stigma were major concerns for families, suggesting a need for public efforts to raise awareness and understanding of autism. The lack of appropriate support and services was also identified as a significant challenge that families face. The study highlights the need for person-centred approaches to service provision, which involves including autistic adolescents and their families in the design and delivery of support and services. The study recommends collaboration among schools, service providers, and families, as well as efforts to promote autism-friendly environments in schools and the broader community.

**Lay abstract:**

The influence of the environment on different groups of people with disabilities has rarely been studied in the context of neurodevelopmental disabilities, such as autism, in adolescence. This study explored how external responses to autism affect the experiences, outcomes and family relationships of autistic adolescents and their family members, including siblings and parents. This study adds to the knowledge of factors that contribute to the social disadvantage and exclusion autistic adolescents and their families face in their communities. Interviews with 30 participants from families with an autistic adolescent show that external factors greatly impact the well-being of autistic adolescents and their families. The school environment, including sensory overload, bullying and negative interactions with teachers, was found to be a key factor that negatively influenced mental health and family relationships. The study also revealed that isolation and stigma are major concerns for families, indicating the importance of public awareness campaigns to increase understanding of autism. In addition, the lack of adequate support and services presented significant challenges for families. The study emphasises the importance of person-centred approaches to providing services, which involve including autistic individuals and their families in designing and delivering support.

## Introduction and background

### Support and care for autistic adolescents

Autistic adolescents usually require ongoing supports from members of their immediate and extended families (i.e. parents, siblings and grandparents as caregivers) throughout their lifetime (Spain et al., 2017). In Australia, the Australian Bureau of Statistics (ABS, 2020) reported that, in 2018, over half (61%) of all people with an autism diagnosis required high support in daily activities and over one third (39%) required support with emotional and social well-being. The 2018 Survey of Disability, Ageing and Carers (SDAC) found that people with an autism diagnosis who needed support (*n* = 170,100) did not receive the support they needed or wanted. More than half (51.7%) indicated they needed greater support than they received. Autistic adults indicated their greatest unmet needs were around cognitive and emotional supports (ABS, 2020).

### Impacts of caregiving on family members

It is well documented that many families who care for or support an autistic family member experience a range of mental health difficulties and stressors (Australian Institute of Health and Welfare, 2019; Baker-Ericzén et al., 2005; Davis & Carter, 2008; Gorlin, 2019; Gorlin et al., 2016; Spain et al., 2017; Tomanik et al., 2004; Trew, 2021). With mothers and fathers as the primary caregivers of autistic adolescents, high levels of depression and anxiety are prevalent in parents caring for an autistic child (Dababnah & Parish, 2016; Dabrowska & Pisula, 2010; Dale et al., 2006; Davis & Carter, 2008; Estes et al., 2009; Gray, 2003; Ludlow et al., 2011; Sharma et al., 2013; Spain et al., 2017) as well as increased levels of stress (Corbett & Simon, 2014; Dumas et al., 1991; Gray, 2006; Gray & Holden, 1992) and fatigue (Cadman, 2012; Firth & Dryer, 2013; Giallo et al., 2013; Hoefman et al., 2014) and increased cortisol levels (Makris et al., 2022). Siblings with an autistic brother or sister are thought to be at greater risk of social behavioural adaptation problems within childhood and into adulthood (Quatrosi et al., 2023). Social behavioural adaptation includes continual alteration and adjustment of an individual’s behaviour that appropriately or suitably responds to another’s behaviour in social exchanges (Gernsbacher, 2006). Social behavioural adaptation problems include a reduced ability to read and respond appropriately to subtle variations in social signalling, including facial expressions and inflection of speech, which when used together and instantaneously can present dynamic, multiple or conflicting messages (Forgeot d’Arc et al., 2020). Concern has been raised for siblings to obtain long-term family care responsibilities from parents (Orsmond et al., 2009; Orsmond & Seltzer, 2007).

### Support for families

Families, including parents and siblings with an autistic adolescent, are an identified group with limited formal and informal support networks who often rely greatly on extended family members for this kind of support (Davis & Gavidia-Payne, 2009; Gardiner & Iarocci, 2015; Luong et al., 2009; Milosevic et al., 2022). External support groups and social participation can be helpful outlets for autistic adolescents and their family members’ emotional and social needs. These groups are considered important for the well-being of families who have an autistic family member (Gregor et al., 2018; Luong et al., 2009; Milosevic et al., 2022); however, barriers to participation exist when various factors, including organisational, emotional and financial issues, prevent families involvement in these (Anderson & Butt, 2018; Brewster & Coleyshaw, 2011; Camm-Crosbie et al., 2019; Griffith et al., 2012; Huang et al., 2022; Liptak et al., 2011; Ryan, 2010), as well as when healthcare settings fail to demonstrate autism-specific knowledge and care (Bosco, 2023; Bradshaw et al., 2021; Nicolaidis et al., 2015). Other factors such as a misalignment with socially acceptable behaviours or cultural norms contribute to a reduction in families’ and adolescents’ social participation and lead further to their social exclusion and isolation via feelings of shame, stigma and embarrassment (Alshaigi et al., 2020; Ng & Ng, 2022; Ryan, 2010; Turnock et al., 2022).

Given the limited formal and informal support networks families have access to and the impacts that autism has on the health and well-being of the autistic adolescent and family members, it is important to consider how external responses to autism (e.g. responses from schools and disability services, and the wider cultural and social responses to autism) might help or hinder families and autistic adolescents.

### Barriers to families’ and autistic adolescents participation in a range of environments

The influence of environments, meaning ‘the physical, social, and attitudinal environment in which people live and conduct their lives’ (World Health Organization, 2007, p. 10), and their role on different groups of people with disabilities, including childhood disabilities, are recognised (Anaby et al., 2013, 2014; Forsyth et al., 2007; King et al., 2013) but infrequently studied in the context of neurodevelopmental disabilities, such as autism, in adolescence (de Schipper et al., 2015; Krieger et al., 2018). Prior research into environmental influences in the context of autism include the familial or parental or peer environment (Orsmond et al., 2006); the physical environment, that is, space, light, smell and noise (Giarelli et al., 2013; Ryan, 2010); the school environment (Hasson et al., 2022; Hebron & Bond, 2017); and community environment, that is, social support or services and broader public (Liptak et al., 2011; Myers et al., 2015) and the participation or involvement of autistic adolescents within these.

Prior research has shown autistic adolescents are impacted across a range of different environments and the effects of this upon their participation and well-being. Studies show that autistic adolescents participate 25% less in cooperative interactions in mainstream schooling and it being a complex and demanding environment for autistic students (Bailey & Baker, 2020; Giarelli et al., 2013; Horgan et al., 2023; Humphrey & Symes, 2011; Paraskevi, 2021; Skafle et al., 2020), and that autistic adolescents are regularly bullied (Eroglu & Kilic, 2020; Horgan et al., 2023; Sreckovic et al., 2014; Zeedyk et al., 2014), experience social anxiety (Jackson et al., 2022; Kuusikko et al., 2008) and breakdowns (Shah, 2019) and score highly on scales of loneliness (Locke et al., 2010).

Other research indicates autistic adolescents have limited peer relationships outside of formal or organised settings (Cameron et al., 2022; Chan et al., 2023; Orsmond et al., 2004) and have low peer acceptance and high peer rejection (Sari et al., 2021); attendance and participation in recreational activities and post-secondary and vocational education are reported to be low (Arnell et al., 2020; Chiang et al., 2012; Lee et al., 2022; Nesbitt, 2000) along with reduced participation in the community (Egilson et al., 2017).

Addressing these issues earlier in an autistic person’s life is crucial as research indicates just over half of the autistic adult population show poor participation outcomes in work, peer–friendship relationships and independent living, and that increasing and strengthening the participation and inclusion of autistic people in childhood might result in increased participation into adulthood (Cameron et al., 2022; Chan et al., 2023; Eaves & Ho, 2008; Egilson et al., 2017; Farley et al., 2009; Howlin et al., 2004; Lawrence et al., 2010; Mason et al., 2021; Turcotte et al., 2015).

Despite this knowledge, scholars are recently turning to autistic young adults to identify the barriers they face to social inclusion and participation in a range of environments (Anderson et al., 2018; Buckley et al., 2020; Goodall, 2018; Mattys, 2018), including in education settings with the experience of autistic individuals shutting down (Hill & Keville Ludlow, 2023; Keville et al., 2021; Shah, 2019) and acknowledged in Australia (Roberts & Webster, 2022). In addition, issues and recommendations around autistic burnout and masking (Mantzalas et al., 2022; Miller et al., 2021; Raymaker et al., 2020) and peer bullying and communication about autism in healthcare settings have been noted (Cappadocia et al., 2012; Eroglu & Kilic, 2020; Jackson et al., 2022; Nicolaidis et al., 2015; Saggers et al., 2011; Zeedyk et al., 2014) and acknowledged in Australia (Bradshaw et al., 2021).

However, at present, there remains limited knowledge to help make sense of the multitude of systemic factors that contribute to the social disadvantage and exclusion autistic adolescents and their families face. To inform interventions and practices for working with autistic adolescents and families, there is an identified need for research to focus on external responses to autism, and on the social and environmental factors that exist outside autistic adolescents and family and their influence on these groups (Kirby et al., 2016; Simpson et al., 2018).

### Research question and approach

As part of a larger study (Trew, 2021), this study incorporates an understanding that the external environment in which families are positioned can influence the family system, including the dynamics, interactions and behaviours within the family, and impact the relationships of family members (Healy, 2005). In response to the literature described above, this study seeks to investigate *what are family members’ perspectives on how external responses to autism impact on their family and on their relationships?* By doing so, this study aims to provide recommendations that help address the external factors that influence the experiences of autistic adolescents and family members and to promote better well-being and quality of life for these individuals and their families.

An ecological systems perspective recognises the interconnectedness between people and their environments, such as an individual’s relationship with their communities, with the broader society and with their family (Bronfenbrenner, 1979, 1989). This perspective can be appropriately applied to families, in that it highlights the significance of understanding actions, conditions and people situated in their system and not separated (Cridland et al., 2014), viewing individuals ‘as interconnected parts of a system that cannot be understood in isolation from one another but as embedded within their family’ (Gardiner & Iarocci, 2012, p. 2178).

Applying a systems lens to the findings focuses the analysis of factors outside the family, to highlight how the health and well-being of individual members in the family and their relationships between one another can be impacted and shaped by the external responses to autism in the broader contextual circumstances of families’ lives.

## Research design

### Methods and study participants

As a part of a larger study (Trew, 2021), this study used a qualitative participatory methodology and phenomenological approach to the research. Phenomenology seeks to understand the world from the participant’s view, and the researcher must ‘bracket out’ their own preconceptions to manage the prejudices and bias of the researcher and ensure they do not impact the data and limit the ‘new’ or ‘fuller’ meaning (Gray, 2009, p. 22).

The focus of this study is drawn from data from semi-structured in-depth interviews with 30 participants from 18 families who were recruited to participate in the research. Family member participants in the study were related to the autistic participants. Participant data included for this study were seven adolescents of 12–19 years of age with an autism spectrum disorder diagnosis, six fathers, 12 mothers and five siblings. Table 1 details these participant groups. Most of these 30 participants identified as White and Australian and less than 10% identified from a Sri Lankan background. All participants resided in Canberra, Australian Capital Territory, which had the highest proportion of people living in relatively advantaged areas (55% in Quintile 5) and the lowest proportion in the most disadvantaged areas (0.7% in Quintile 1) (ABS, 2018).

**Table 1.** Participant groups, numbers of participants and gender of participants.

Participant groups	Adolescents with an autism diagnosis aged 12–19 years	Siblings	Mothers	Fathers
Number of participants	7	5	12	6
Gender of participants	3 females 4 males	4 females 1 male	12 females	6 males
Ethnicity of participants	White and Australian	White and Australian and Sri Lankan	White and Australian and Sri Lankan	White and Australian
Socioeconomic status of participants	Quintile 5	Quintile 5	Quintile 5	Quintile 5

The sampling approach was designed to engage with families and to connect and collaborate with individual members. The project’s recruitment strategy emphasised fostering constructive connections and relationships with Canberra-based autism support services, local educational institutions and regional youth and family disability assistance services. To recruit participants, the research was promoted via the newsletters and bulletins of the services and schools. No restrictions on family structure were imposed on the sampling plan. Family members were those considered as ‘mother’, ‘father’, ‘child’, ‘brother’, ‘sister’, ‘husband’, ‘wife’ or ‘partner’, and definitions were to be determined by the family members themselves.

To be eligible for this study, participants needed to have firsthand experience of autism in a family context, be open to discussing family and autism, and consent to taking part in an initial interview with a potential follow-up interview. Autistic participants eligible for this study included those with a reported diagnosis of autism spectrum disorder and included those with an intellectual disability or other co-occurring diagnosis, for example, attention deficit hyperactivity disorder. Autistic participants were provided with choice as to (a) where the interview was conducted, for example, in a public or home setting; (b) how the interview was conducted, for example, in person or online; and (c) whether they participated in interviews independently or with a support person present, for example, a parent, carer, guardian or disability/service worker. In addition, participants were required to allow the researcher to audio record and/or document the interviews and to use the data for publication in a dissertation and other related publications.

### Data collection and analysis

Data for analysis were obtained from the audio-recorded, semi-structured in-depth interviews conducted with participants with the aid of an interview guide. The interview guide lists broad, open-ended questions on topics related to the research question; these questions were asked of all participants but some prompts specific to the participant group differed. Examples of the questions used to guide the interview are provided in Appendix 1. The topics covered in participant interviews were family relationships, family interactions and communications, family identity, positives and strengths of being a family and services families accessed. Interviews were held in-person with all participants. Most participants chose to be interviewed in their home and were interviewed individually. Just two participants, a mother and a sibling, chose to have a family member present during their interview.

Data collected were managed using the NVivo 12 Plus programme, which is software designed to collect, store and analyse unstructured and non-numerical data. Data were analysed using a thematic method of analysis. Thematic analysis (TA) is a qualitative method that can be applied across a range of epistemologies, interpretive frameworks, and research questions (Braun & Clarke, 2014; Charmaz, 2006; Lorelli et al., 2017; Nowell et al., 2017); it is used to recognise, analyse, organise, label and report on themes produced from data sets (Braun & Clarke, 2006). TA provides systemisation to textual data and enables a deep and rich exploration of patterns within a data set (Attride-Stirling, 2001). A process to ensure rigorous coding was used which included the development and peer review of the codes by the research supervisory team. This process checked that data had not been misinterpreted, resolved discrepancies in data coding and that researcher bias was addressed. The research team read through the data multiple times, verifying the codes and themes for reliability and validity.

Family systems theory (Cox & Paley, 1997; Minuchin, 1985) informed the approach and design of the study, recognising that family experiences are shaped and influenced by a range of environmental system levels, including interactions among family members and interactions with community, school, peers, other families and workplaces, and with the service provision sector. This perspective helped to provide a contextual understanding of the presentation and analysis of the data and informed the interpretation of the findings. The perspective underpinned the consideration of the contextual circumstances in which families are positioned when developing and framing the implications of the research. The participants’ insights and experiences are presented in the findings section, using illustrative quotations supported with pseudonym ascribed participant quotes.

### Ethics approval

This study was approved by the Australian Catholic University (ACU) National Human Research Ethics Committee (HREC), study registration number 2019-33H.

### Consent

This study ensured that obtaining consent from participants was an ongoing process that was frequently reviewed and updated. Participants were given multiple opportunities to confirm or retract their consent to participate in the interviews. To ensure that participants fully understood the nature and extent of this study, they were provided with information and asked to consent through a tick-box process. Participants were also reminded of their right to withdraw from this study at any time without fear of repercussions, and that their involvement in this study would not affect any of their existing support services. For individuals below the age of 18, parental consent was mandatory, and a separate tick-box form was used to obtain the child’s assent. The consent and assent forms were kept on record, and the researcher recorded the discussion to document the informed consent process.

### Community involvement

Two families each with an autistic adolescent and as a part of an advisory group contributed to the development of this study and provided valuable insights. To gauge the potential interest of the local community, autism support organisations in the Canberra region were approached and given information about the project and the advisory role. Each family and all the members in the families, that is, mothers, fathers, siblings and the autistic adolescents participated in various research tasks. These included interview methods for autistic adolescents, interview schedule revisions, interview rehearsals/pilot tests, workshopping potential ethical concerns, sensitivities and other practical aspects of involving autistic adolescents in interviews, as well as verifying transcripts and results.

## Findings

The findings presented here examine family members’ perspectives on how external responses to autism (e.g. responses from schools and disability services, and the wider cultural and social responses to autism) impact them and their family relationships. Central to this is the observation that autism is interwoven and interconnected with many aspects of families’ lives. Together, these external responses to autism draw attention to the influence the environment has on families’, the impacts this has on their family relationships and how families with an autistic family member are made to feel different to other families because of external responses to autism. Families’ insights and experiences are presented in detail, using illustrative quotations throughout the global theme of *Made to feel different*.

### Global theme: made to feel different

Families report that school and disability service organisations’ responses to autism, both the positive factors and the stress and the pressures that occur in these settings, flow into families’ homes and impact family relationships and family members’ well-being. Broader external responses to autism, including disability policy, funding, and the structure of services and institutions that family’s access, isolate families from their communities and indirectly impact family relationships. Pressures and expectations of cultural and social ideals also enter families’ homes to make them feel different to other families, reinforcing their sense of isolation.

**Figure 1.**
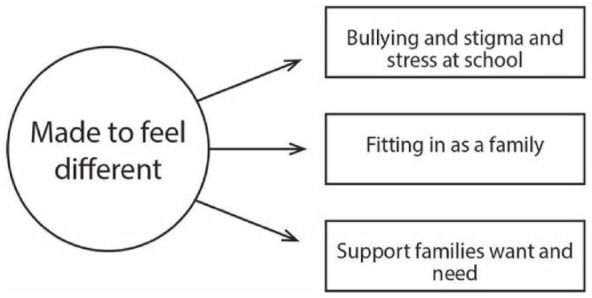
Network map of external responses to autism global and organising themes.

Families react to these external responses to autism, and they highlighted in interviews the kinds of supports they want and need to combat some of the challenges they face. These supports include health professionals such as a social worker or psychologist for autistic adolescents, support groups for parents and mentor support groups for siblings. Families need these supports to create meaningful and positive family experiences, which, in turn, promote strong family relationships. Families commented on the types of supports that could be offered but that were not available or easily accessible at the time of their interviews. Figure 1 illustrates organising themes that were constructed within this global theme.

#### Bullying and stigma and stress at school

The experience of school for autistic adolescents was observed to have an impact on the relationships at home for all families. At the time of their interviews, many relied on school as a main source of support as they waited to join and access funds through the National Disability Insurance Scheme [NDIS]. The NDIS enables families to access supports without using their own funds. Families described a range of factors that contributed to a poor school experience, such as the school environment, particularly classrooms.

##### School classrooms

Some parents described classrooms as difficult, busy and noisy places with high amounts of sensory stimulation for their autistic child. All families understood the school environment as a place that was, in general, not suited for autistic adolescents. Noise was a major factor that parents identified as an issue for their autistic children: ‘I actually feel it’s a noise issue … because all the kids coming in, yelling, screaming, and moving the furniture, and crashing into him’ (Nairi, mother). Once home, after school finished for the day, some parents reported their autistic person shutting down or melting down, as a release after holding in their stress and emotions. The home environment was a place adolescents could release the stress of school:

Corey (father): They’d come home from school and be completely shut down … we have a lot of behavioural issues where they’ve been rather violent … everyone finds it hard to believe that they will misbehave, because they’re perfect at school. But they’re perfect at school because they pin it all up inside, then [it] unleashes when they get home.

Helen (mother): He just couldn’t cope with the classroom setting … keep up and function … the social stuff to deal with … the noise to deal with. All the things that make a classroom so difficult … he was not functioning in the classroom. He was just shutting himself down, blocking everything out. … He gets home, and all hell breaks loose.

As well as noise, parents understood classrooms to be unstructured and disruptive settings for adolescents. For example, Corey (father) said, ‘I think a lot of it is the sensory overload, it comes from just being in disruptive classes’. Adam (father) had similar observations: ‘The school classroom we realised was such a busy environment … Kids are sitting on the floor, there’s things fluttering all over. There’s lots of handing out of papers and lots of meaningless talk and discussion’.

##### Peers

Autistic adolescents felt stigmatised and were bullied by peers at school. Stigmatisation and bullying occurred in both primary and high schools. Lucy (autistic person) reported feeling uncomfortable talking about autism with friends, and felt awkward when peers would use the word ‘autistic’ to describe someone or something in a negative way:

Lucy: Only one person really knows that I have autism. Most of my friends are aware of it, but we don’t talk about it … I don’t really feel comfortable talking about it. I don’t really feel like I belong when I say that … I’ll feel really awkward … Lots of boys, for example, in the year, will joke that something is autistic … I’m glad it is never any of my friends.

Siblings of autistic adolescents reported feeling uncomfortable disclosing their autism diagnosis to peers, and parents were aware of this discomfort. For example, Stephanie (mother) said, ‘They’d seen how his sibling had been treated over the years, they were worried that that might apply to them’. Possible repercussions if siblings did talk about having an autistic brother or sisters were mentioned. For example, Luke (sibling) noted that ‘people will act differently around you, treat you differently. It’d be a lot more difficult to do things’.

Jim (autistic person) recalled other autistic students being bullied at their school because of how they acted: ‘I remember boys from my old school who had it [autism] quite a bit worse, and they were picked on because they acted different’. Many parents confirmed the bullying and teasing these autistic adolescents encountered at school:

Cassie (mother): They’re getting a lot of negative stuff because of it, so as far as I understand, autistic is an insult you use in the playground to label other children. It’s now ‘the’ [go to] word. If you do something inappropriate or socially out there, they’ll say, ‘What’s wrong with you? Are you, autistic?’

Helen (mother) reported the challenge of primary school for her autistic person due to stigma and bullying:

Helen: Primary school overall was very challenging for them. They were bullied a bit in the early childhood centre. Then when they went to primary school and they saw the Learning Support Units, and they saw the other kids there and they saw the other kids teasing them. They made a very conscious decision they didn’t want anybody to know that they had autism.

Bullying and stigma not only affected autistic adolescents but also affected their siblings. Sibling Eileen reported feeling uncomfortable talking about her brother’s autism diagnosis at their current school because other students made jokes about autism:

Eileen: I don’t really feel comfortable talking about it in school because people who you know make jokes about it [autism] … Not necessarily my friends, but saying, ‘Oh, you’re such a retard’ to people. Or ‘You’re autistic’. It’s like a joke. Or ‘I’m so autistic’, because it’s like, I’m weird or something.

Some siblings at school had to take on a caring role outside of the family home. Amandi was made to feel different when peers made comments about her autistic brother:

Amandi (sibling): [There have been] multiple instances where I was trying to make my own friends, have my own lunchtime, and anytime he had a tantrum or breakdown they’d [peers would] be like, ‘Oh, your brothers in this part of the school’. I’d have to go find him and then look after him … I guess the way I realised that it was different was because everyone else made it obvious.

##### Teachers

A small number of autistic adolescents experienced hostile and negative interactions with teachers. Teachers were described as having a negative impact on the well-being of a small number of the autistic adolescents. These teachers overall were described as people who did ‘not listen’, were ‘unforgiving’ and who ‘shouted’ and ‘got angry’. Lance shared that ‘the teachers that were bad were teachers that would shout at me and get extremely angry. I had a couple of teachers who were quite physically forceful. That was very unpleasant’. Lance reported what he remembered most about teachers were how ‘authoritative’ they were:

Lance: The teachers were always, in my point of view as a little kid growing up, an oppressive force … what I remember most of all about teachers that really hurt me as a little kid, was how authoritative they were.

Parents who repeatedly tried to interact with teachers throughout their child’s schooling described teachers who had no skillsets for understanding or managing their autistic adolescents. Stephanie (mother) said, ‘It’s that lack of understanding that the teacher has which is the critical factor … If you don’t understand why I’m behaving the way I am’.

Rob, Dwayne and Adam (fathers) shared their experience of teachers at schools. Their accounts are provided below, in turn:

Rob: They had no skill set for understanding or managing children. [My child] didn’t fit into a normal box. They’re very limited in what they can offer.

Dwayne: The schools need to do more. They need to be more flexible and be prepared to learn new techniques.

Adam: Most teachers had not heard the term Asperger’s syndrome. Their idea of autism was the Rain Man stereotype. They had no idea about what the spectrum of behaviour might be let alone how to intervene for kids on the spectrum.

Some parents commented on teachers who did not understand autistic adolescents. This also included some teachers’ limited sensitivity to and awareness of the needs of autistic adolescents:

Adam (father): There were a few unfortunate events where [my child] was made to feel victimised and stigmatised. It was one class trip. [My child] was told they couldn’t go because the school just said, ‘Well they’ve got Asperger’s syndrome. They might run off. They might act out. They might do something, so therefore they can’t go’.

##### School and isolation

Because of adolescents’ negative experiences at school, four families in the study had chosen to remove their autistic person from school, and home schooled them. For these families who experienced great challenges with the school system, parents felt they had no other option but to remove their autistic person from school and to homeschool them. These parents had no formal supports: ‘That’s probably the biggest area that’s hard for us is not having enough support in school’ (Francene, mother). Adam (father) described his son’s increased anger at both school and home:

Adam (father): He was exhibiting some quite angry behaviours at home. They were angry at school. They were angry at home. They were unhappy … in a bad, negative space … I home schooled, which was tough because they weren’t the most willing pupil. They were still carrying a lot of anger. They sometimes were oppositional. But I couldn’t do that forever because I think the home-schooling gig is a difficult one. If you are carrying too much emotional baggage you can’t achieve that level of emotional detachment that you need to teach a child.

Lance, an autistic adolescent who home schooled identified both positive and negative aspects of one-to-one tuition in the home environment with a parent, but were also conscious of the demands and impacts this had on their relationship with their parent:

Lance: Part of it is that I do one-to-one tuition, which is really good for me. But also, the main thing I’ve noticed is I was left with no real knowledge [of] how to complete tasks because completing tasks, doing work, organising my time, schools managed to stigmatise that so much that I associated it with nagging and fighting with my parents and misery. … I believe this is a struggle for many parents, especially with kids on the spectrum.

From parent reports and some autistic adolescents, many other autistic adolescents in this study had at some point in their schooling taken long periods of leave or absences from school due to the school environment’s impacts on their well-being and mental health. For example, Jim and Rory (autistic adolescents) shared,

Jim: At my old school I got bullied since the first year. I don’t think we’d even got past the first term, and I started getting picked on. I told the teacher. I said, ‘Stop, I don’t like it’. Then I said, ‘Bugger off’. Then language just got worse, I told the teachers, did all the things I was meant to, and nothing really happened.

**Rory:** I just see it [autism] as a label, something to stress about, something to keep secret. Because, every year, I’ve had to change schools. I’ve never stayed in one place … There was always some reason … probably mistreated a bit … I think it’s more of, I don’t know, a curse or something … It’s just a bit hard making good friends that stick around for a while … people will act differently around you, treat you differently.

Poor experiences at school left some autistic adolescents reliant on their parents for increased emotional and social supports, as well as friendship. Home schooled, Lance (autistic adolescent) described the friendship his father provided to him and the importance of the friend role in their relationship:

Lance: Probably one of the main things they give me is friendship, especially my dad. I don’t have many friends, especially since I left school because I didn’t really rely on friend groups or friend networks.

A couple of mothers reported on the challenging school experiences for their child and the impact this had on themselves and their families isolation:

Karen (mother): High school’s been a lot harder … a major factor is [my child] hasn’t made close enduring friendships from school … You do tend to feel a bit, ‘trapped here being the mother of a 15-year-old who’s totally dependent, and I don’t have a life’ … It just tends to reinforce a sense of being very isolated.

Janette (mother): I think during the summer holidays is the time where it’s really easy to just think of ourselves as, ‘We’re just a family’. Then you hit school, and you hit all the problems that school entails for them, and you go, ‘Oh yeah, we’re not just another family. It’s really hard’.

#### Fitting in as a family

Families’ absence from school, or the limited interactions they had with others at school, contributed to feelings of isolation. All mothers of autistic adolescents described the isolation they experienced during these school years. Cindy (mother) reflected on the difference between her primary school and high school experience: ‘[High school] is a little bit alienating. I don’t think we put ourselves out there nearly as much as we did [in Primary school] … I think you do start living a more insular life in a way’. Janette (mother) said, ‘There’s lots of conversations at school around the kids and it’s very hard to interject with your child when they’re so different. No one really can understand that’.

Over time, families became aware of the differences in their lives compared with other families without an autistic member/s. Dennis (father) reflected, ‘There was a development of an awareness that our life path was going to be quite different … a special needs kind of existence … key elements of existence that seem so autistic that you couldn’t really ignore that’. Stephanie (mother) reported that cultural norms emphasised their family’s sense of feeling different from other families, and highlighted the need to fit in with social expectations:

Stephanie (mother): To show them how to operate in the neurotypical world, are you basically rejecting who and what they really are? Walking that fine line of saying, ‘I really love you and I think you’re fantastic and special to me, but you know, you need to fit in here’.

For families like Stephanie’s, trying to fit in and function within social norms and expectations meant not drawing unnecessary attention towards themselves. This was an important consideration for families when they left the home to go out into the community:

Monica (mother): We go for the small wins and celebrate when they happen. For us, to go out into the community and return home without calling attention to ourselves, to fit in, is a positive experience and seen as a huge win.

In the community, families received negative comments and judgement from others when their autistic child became overwhelmed with the sensory input of the environment. Nairi (mother) said, ‘I got so sick of trying to explain, “He’s not a naughty boy. He’s just got sensory issues and cannot cope”’. Maya (mother) recalled, ‘I can remember when he was quite young and he was having tantrums at shops, I’ve got stares from people, people were staring at us and that annoyed me. I felt judged’.

Parallel to families’ experiences, the media’s portrayal of families who lived with autism as ‘super families’ (Monica, mother) who overcame autism through a lived experience of ‘tragedy, persistence, then triumph’ (Dennis, father) left many families further isolated from the world outside the family. These families described being caught between ‘a special needs existence’ or ‘disability world’ (James, father) and the mainstream community’s needs and wanting to fit in with these.

Parents commented on the stereotypes and misconceptions about autism that they had faced in the community. Karen (mother) explained, ‘There are lots of wrong ideas, people with autism are cold, unemotional, lacking in empathy. My daughter is such a warm, loving person’. Health professionals, including general practitioners and paediatricians, were some of the groups that provided inaccurate information about the condition. Margaret (mother) commented, ‘The doctor said, “Oh, autistic kids don’t make friends”’. Janette (mother) had a similar experience, commenting that ‘the paediatrician had very strong views . . . things like autistic children just sit in the corner and rock’.

Parents challenged some of these views with examples from their families. Janette (mother) explained, ‘The stereotype that a child can’t show you love or affection. Obviously, I knew that Jake was affectionate … there’s a more nuanced understanding around the spectrum and children, they do show their family love’. Reflecting on the difficulties autistic adolescents experienced, Lance (autistic person) commented on how autistic people are defined by others primarily by their difficulties:

Lance: Apart from very intense interests, I think a lot of the time [people with Asperger’s syndrome] are way more easily defined by difficulties. They’re way more easily defined by things that people struggle with, anxiety, depression, trouble talking to people, expressing oneself, and dealing with certain situations.

Lance reflected on the experience his parents might have had when raising him: ‘They may not have had an enjoyable experience raising me because they were told there was going to be so many difficulties’.

#### Support families and autistic adolescents want and need

Autistic adolescents commented on the value of being connected with a health professional, including a social worker or psychologist who helped adolescents express themselves emotionally, as well as helping them socially. This helped to create or influence positive impacts on family relationships:

Lucy: If I have any problems, she helps me, shows me, and finds a way. She talks to me and shows me ways and skills, how to exit conversations better. She teaches me friendship skills, social skills. It gives me a chance to escape and express my emotions. It then helps me in life.

Most autistic adolescents identified several main qualities they valued in their support person. The person was described as someone they could talk to, who ‘actually listens’ and ‘actually helps’ solve problems, and who taught them skills, including friendship skills and social skills, and how to be less stressed. Autistic adolescents also identified benefits of seeing a support person, which included ‘a chance to escape myself’, ‘a chance to have some time to myself and do stuff I want’, and ‘an outlet and avenue to express emotions’. Ken shared that his support people of choice were ‘psychologists, because I know that it’s been good for me to get stuff off my mind’.

As well as wanting and needing to be listened to and supported in their family relationships, autistic adolescents stated the importance of service providers having a greater understanding and more knowledge of autism in families, and of how families themselves perceive and experience autism. Adolescents wanted service providers and professionals to understand that each autistic person is a distinct individual: ‘People are saying that everyone on the spectrum’s the same, but that’s not true. People on the spectrum are a bit different and the needs of people on the spectrum aren’t the same’ (Nina, autistic person). All families agreed that this gap could be addressed if organisations employed autistic people and their family members:

Joanne (autistic person): Most of the staff should at least be on the spectrum, or least somewhat neurodivergent. It’s important so that the people in [the support service] know what kind of things to expect and what kinds of things to do with these families from their own experience.

Monica (mother): When organisations are hiring, they shouldn’t be hiring people that have degrees and no experience in disability. Because they haven’t lived it. They should be hiring people that have lived it. Understand it. Have seen all the different shades and flavours.

Autistic adolescents recognised the importance of individualised supports for themselves and their families:

Jim (autistic person): I would say probably try to get people, adults, and children, who are on the spectrum, to talk about their experiences, and what they struggled with within the family, and what they found supportive within the family. Families can take that on from the autism perspective … Either the people with autism from the family or not, talking about how growing up was for them, in whatever environment they had, and how it could have been improved, and how what’s really beneficial for them being a family.

Some autistic adolescents and some siblings recognised the need for supports groups for parents:

Lance Fostering connection between(autistic person): parents I think would be a good thing. Parents not feeling like they’re alone and feeling like they can talk to others, but also not feeling like they’re just being jammed in with people who are dealing with a completely different struggle.

Kate (sibling): [Support] for the parents, a group to get together to talk about the difficulties of parenting an autistic child, because I think they probably are the ones that struggle the most … I think that would be beneficial, to work together, not only as a parent with a child that has autism but working together as parents . . . Mum and Dad take very different approaches when it comes to [my brother]. Obviously, they’re both doing their best, but I think communication with the child, and then communication with each other, would probably be a really good service.

These supports suggested by Lance and Kate might help parents to feel more connected to each other and united in their approach to parenting.

Parents, in considering the supports they needed, suggested that service providers could have longer operating hours and flexible practice models: ‘Being more flexible on operating hours would be helpful. Working families find it so hard to meet our children’s needs, let alone try to get time for fun outings’ (Francene, mother). Cassie (mother) explained that parents appreciated services that visited the family home, as this meant the family did not have to ‘juggle’:

Cassie: Because most parents are flustered … The exhaustion’s a constant thing and everyone I’ve spoken to is physically drained … I’ve found it much easier to do things when people come to us. I think most parents really appreciate services that come into their home because they don’t have to juggle … If more services are helping with the day-to-day stuff, or even someone taking all the kids out to an outing … more intensive supports.

These supports suggested by Francene and Cassie could mean families have more choice and flexibility in how they spend time together.

Supports identified by siblings included mentor support groups, to help reduce isolation and caring responsibilities for siblings, and supports to address sibling conflict and arguing. Siblings wanted to get help in coping with arguments that occurred daily:

Amandi (sibling): I wanted a sibling group because it’s [caring is] very weird and I would have very much appreciated if someone, growing up, told me, ‘Hey, it’s OK’ … Cause it’s very different talking to someone about caring who has no idea what’s going on … I never had anyone to talk to about that stuff … maybe I wouldn’t have felt so isolated or like an outlier … it would be nice to have that sort of commonality between me and someone else.

Sarah (sibling): First, people could ask them [siblings] what it’s like at home for them, getting to know the family. I would ask them, are there many arguments at home? … I would help with that. Because I know arguments are a really big thing in this house … like I said before we cannot go one day without an argument.

## Discussion

This study aimed to explore the experiences of autistic adolescents and their families, with a particular focus on the external factors influencing their lives and family relationships. The findings considered the wider context outside of the family as a unit and highlighted the interplay between the external environment and the well-being of autistic individuals and their families.

Among the different participant groups (parents, siblings and autistic adolescents) in this study, the themes highlight an overall interconnectedness and similarity of experience as to how external responses to autism from school, media, service providers, and professionals, and broader social norms and values, influenced individual participants and families and impacted their relationships. This included families who struggled to fit into the community, who felt rejected from their schools and peers and who did not have access to suitable supports. These factors led to increased challenging behaviours displayed, and acknowledged by the autistic adolescents, including physical, emotional and social behaviours such as anxiety, overstimulation and anger. Jointly, these factors draw attention to the importance of being aware of the setting created by the physical and social and attitudinal environments, the influences these have on autistic adolescents and family members and the impacts had on the family unit and on the health and well-being and relationships of its members.

The findings emphasise the need for a multifaceted approach to address the multiple systemic challenges families face. This story is interconnected, as this study recognises, with the exploration of the experience autistic individuals and their families face in various environments with a range of services and in the wider community.

### Education settings

Only recently, research has begun to address the struggles inherent within society and the more external and environmental factors (beyond families and the individual) which increase the vulnerability of autistic individuals and their families. In the United Kingdom, Bronfenbrenner’s bio-ecosystemic theory has been applied to understanding the experiences of autistic individuals and their families face in education, acknowledging that this is part of a wider issue. This systemic focus was initially recognised by Hebron and Bond (2017) and recently Hasson et al. (2022). These highlight the struggles families face managing external educational factors for autistic children and adolescents in mainstream provision.

In this study, schools greatly impacted the families, as they faced extended wait times for funding and inadequate support from schools. In school settings, autistic adolescents experienced exclusion and struggled in high sensory environments. As such, families emphasised the importance of receiving adequate support from schools to ensure the success and well-being of their autistic members. This includes appropriate accommodations, sensitivity to sensory needs and the implementation of strategies to prevent bullying and stigmatisation.

In the United Kingdom, the value of home-schooling, reducing sensory demands in education settings and the experience of shutting down has been noted previously (Giarelli et al., 2013; Hill & Keville Ludlow, 2023; Keville et al., 2021; Shah, 2019; Skafle et al., 2020), and acknowledged within Australia (Roberts & Webster, 2022). In addition, issues and recommendations around bullying and communication about autism have been noted previously (Carrington et al., 2017; Eroglu & Kilic, 2020; Jackson et al., 2022; Zeedyk et al., 2014) and support this study’s findings. These suggest that training for educators about autism might help towards the provision of inclusive and supportive learning environments for autistic individuals in school. Schools could collaborate with parents to implement appropriate accommodations and modifications for autistic students, such as sensory-friendly classrooms and individualised support plans. In addition, greater advocacy for inclusion in schools to reduce stigma and bullying and to encourage positive peer relationships for autistic students is needed.

### Health care and service provider settings

This study highlights the challenges faced by families with autistic adolescents due to a lack of access to suitable formal and professional supports and affordable respite options. The findings show that the families lack access to suitable supports and experience powerlessness and exclusion from these settings. Perspectives from parents, siblings and autistic adolescents revealed the need for tailored assistance for caregiving.

The findings from this study reflect those from other studies (Read & Schofield, 2010; Roughan et al., 2017) which have suggested that many health services are failing families with autistic children and adolescents and others note several barriers that prevent their participation in these (Brewster & Coleyshaw, 2011; Liptak et al., 2011; Ryan, 2010). Some authors are calling for organisations and service providers to develop innovative interventions to meet the needs of this diverse client group (Gabovitch & Curtin, 2009; Hodgetts, 2013; Hodgetts et al., 2015; McKenzie et al., 2020; Nicholas et al., 2020; Roughan et al., 2017; Wilson, 2013). In these studies, and the current study, parents recognise a lack of appropriate family-centred care in services, including limited understanding of what services and supports families are receiving. Parents of autistic children in prior research report that formal services are not meeting their needs, which contributes to their feelings of isolation and alienation (Galpin et al., 2018).

This study also found that health professionals often lacked understanding of autism, which contributed to families feeling powerless and excluded. In this study, autistic adolescents, however, expressed the importance of being connected with health professionals, such as a social worker or a psychologist who understand autism and can provide emotional and social support. These professionals, as suggested by the autistic adolescents, can help them to express themselves emotionally and navigate social situations, leading to improved relationships and social participation outcomes.

From this study, a recommendation is for health professionals to update their understanding of autism to provide better support for autistic individuals and their families. A need for autistic-led communication about autism in healthcare settings has been noted (Bosco, 2023; Bradshaw et al., 2021; Nicolaidis et al., 2015) and training programmes for specialist autism healthcare professionals are suggested to focus on current community perspectives of autism and the unique experiences of autistic people (Milosevic et al., 2022). Collaboration among health professionals, autism experts, the autistic community and those with lived experience of autism could help bridge this knowledge gap.

### Parent and autistic adolescent support groups and voluntary or paraprofessional settings

In this study, families identified the need for informal or non-professional support groups for both non-autistic parents and children to develop and nurture connections with each other. These groups are suggested to provide a safe space for family members to share their experiences, learn from one another and develop a sense of belonging. Access to and the availability of appropriate and tailored support groups for parents, siblings and autistic adolescents are vital and considered fundamental for future autistic adults’ participation in a range of environments and settings (Cameron et al., 2022; Chan et al., 2023; Mason et al., 2021) and research highlights how autistic adults struggle for appropriate services (Anderson & Butt, 2018; Camm-Crosbie et al., 2019; Griffith et al., 2012; Huang et al., 2022).

From the findings in this study, these kinds of support groups are suggested to focus on specific challenges faced by each family member group and aim to reduce isolation and encourage positive family and community connections. Prior research supports this call by families as these groups are perceived by parents of autistic children as more useful than formal or general health care and disability service provider settings (Milosevic et al., 2022).

In addition, focus groups and paraprofessional autism networks have been shown to provide meaningful and positive experiences for families, with parents reporting that swapping family stories in an open forum created ‘meaningful moments’, bringing individuals closer together (Loukas et al., 2015, p. 461). In addition, support networks in the community can increase knowledge, introduce and teach improved living patterns, increase capacity for heightened coping skills and help bring desired changes while helping families to know that they are not alone (Lock et al., 2013; Murphy & Verden, 2013; Nieto et al., 2014). More recently, undergraduate student volunteering groups as a support for parents with an autistic child and for autistic children and young people has shown benefits for families and autistic people’s well-being and general health (Breithaupt et al., 2017; Jominol et al., 2022; Marggraff & Constantino, 2018).

Prior research has identified the importance of ‘place making’ (Loukas et al., 2015, p. 461), which involves creating a safe space for families and parents to talk about their autism life experiences; providing such a safe space has been found to create positive interpersonal changes among families and their autistic member (Loukas et al., 2015; Ludlow et al., 2011; McCabe & Barnes, 2012; Safe et al., 2012; Zhang et al., 2013). However, many parents regard support groups and social groups as inaccessible, partly because of professionals’ lack of understanding of and limited involvement in the unique ways in which each family functions, and because these services are either lacking or communication about them is inadequate (Khan et al., 2016; Milosevic et al., 2022).

### Community settings

In this study, a broader lack of autism knowledge existed within the community, resulting in negative responses and social isolation for these families. This isolation increased pressure on family relationships and contributed to a negative family identity. This was for all families despite living in advantaged areas. In addition, families could not access the necessary support, which contributed to negative relationship and well-being impacts.

Prior research identifies that support for autistic children and their families varies widely and is not accessed equitably (Milosevic et al., 2022). However, further research is needed to identify which families are most at risk of experiencing negative effects from inequitable service access and provision of supports. The experience of stigma by autistic people and their families is relatively understudied (Alshaigi et al., 2020; Grinker, 2020; Liao et al., 2019; Ng & Ng, 2022; Turnock et al., 2022), despite the evidence that stigma contributes to poor outcomes across a range of domains and impacts on an autistic person’s overall well-being (Turnock et al., 2022). More research, however, is needed to identify and understand the interplay between dimensions of stigma, collective efficacy and advocacy communication among autistic people and their families (Farsinejad et al., 2022; Grinker, 2020; Leadbitter et al., 2021; Turnock et al., 2022).

From the current study findings, to reduce families’ feelings of isolation and promote inclusion, communities including the broader public need further education about autism. This is suggested to be fostering an understanding and empathy among the public and extinguishing uninformed stereotypes and misconceptions about autism. Prior research shows how stereotyping negatively impacts autistic people and their families and other neurodiverse communities (Liao et al., 2019; Ressa & Goldstein, 2021).

Community-wide awareness campaigns and educational programmes are suggested by participants in the current study to help reduce stigma and promote understanding of autism. The findings suggest encouraging a strengths-based perspective on autism and neurodiversity to foster more inclusive communities and enhance the overall well-being of autistic people and families. This suggestion has some support from prior research conducted in the United States, of a nationwide campaign to increase autism acceptance among the public and general community which invoked positive changes in the general parent community (Anthony et al., 2020). Further research is needed beyond general awareness campaigns to measure actual behaviour change among the public and in family communities.

### Study limitations and future research

Recognising the study’s limitations is important, such as the reliance on self-reported data and the possibility of participant recall bias. Moreover, the research concentrated on a relatively limited number of families, including gender and ethnicity and socioeconomic status, which may not represent the wider population of young autistic individuals and their families. Further studies should utilise larger and more varied samples, as well as implement longitudinal designs to investigate the ongoing effects of external factors on family relationships and well-being for families with an autistic member.

Future and ongoing research is needed to explore further the multiple systemic challenges autistic people and their families face. As this is a global issue, bringing that global story together will facilitate much needed change at a global level. This will also include explicitly bringing together studies of the socio-cultural factors that impact family well-being for autistic individuals and their families. Bringing studies together which build on this and present this wider story will only enhance the evidence base and implementation of recommendations for the benefit of autistic individuals and their families.

## Appendix 1

### Interview guide

#### Identity

1. In what contexts would or wouldn’t you use the term ‘autism’?
2. How do you talk about autism with your family?
3. How do you talk about autism to people outside the family?
4. In what ways does autism make your family unique or distinct?
5. How does autism play a role in who your family is?
6. In what ways does your family identify with autism?
7. What do you think about the label ‘autism?’

Questions for young person on the autism spectrum

a) In what ways do you identify with the term ‘autism’? (What does autism mean to you?)
b) In what ways does autism make you unique or distinct?

#### Relationships

8. How do you all get on as a family? Do the children get along well with one another? Do the children get along well with the parents?
9. In what ways does autism influence family relationships? Positive and negative influences?

#### Interactions

10. What does your family do together?
11. In what ways does autism influence what your family does together?
12. In what ways does autism influence family interactions? Positive and negative influences?
13. Does your family have some respite or relaxation time? What do you do during this time?

#### Services and supports

14. What supports and/or services does your family currently receive/access?
15. What are your support needs as a family?
16. In what ways do services fit with your family identity, relationships and interactions?
17. In what ways do services meet your needs as a family?
18. Could you tell me about any unmet support needs?
19. In what ways can these services better support your family?
20. If a new service was created for families with a young person on the autism spectrum, how should it provide support?

#### Finishing up: the future

21. Thinking about your family’s future, what would help to reach your hopes and expectations?
22. Is there anything else you would like to mention regarding your family identity, relationships and interactions, something I haven’t asked you about?
